# Acupuncture and related techniques for type 2 diabetes mellitus: A systematic review protocol

**DOI:** 10.1097/MD.0000000000014059

**Published:** 2019-01-11

**Authors:** Meilu Liu, Jianrong Chen, Qing Ren, Weifeng Zhu, Dongmei Yan, Heyun Nie, Xiaofan Chen, Xu Zhou

**Affiliations:** a Evidence-based Medicine Research Center, Jiangxi University of Traditional Chinese Medicine, Nanchang, Jiangxi; b Department of Endocrinology, The Second Affiliated Hospital, Chongqing Medical University, Chongqing; c School of Chinese Medicine, Hong Kong Baptist University, Hong Kong SAR, China.

**Keywords:** acupuncture, protocol, systematic review, type 2 diabetes mellitus

## Abstract

**Background:**

Type 2 diabetes mellitus (T2DM) is a major global health problem. As a complementary treatment, acupuncture and related techniques are widely used to treat metabolic and endocrine diseases, but their efficacy and safety for T2DM are yet to be established. This systematic review will qualitatively and quantitatively summarize the current randomized controlled trial (RCT) evidence regarding the efficacy and safety of acupuncture and related techniques in patients with T2DM.

**Methods:**

Comprehensive literature searches will be performed on PubMed, Embase, Cochrane Central Register of Controlled Trials, and a trial registry “ClinicalTrials.gov” from inception to December 3, 2018. We will include RCTs for patients with T2DM that compared acupuncture with placebo, antidiabetic drugs, lifestyle interventions, or the combination. Primary outcomes are fasting blood glucose and hemoglobin A1c. Secondary outcomes include 2-hour blood glucose, fasting insulin, homeostatic model assessment for insulin resistance, incidence of diabetic complications, and acupuncture-related adverse events. The risk of bias of the RCTs included in the review will be examined using a revised Cochrane handbook tool. Heterogeneity will be detected using Cochran *Q* test and *I*^2^ statistics. With the use of random effects model, we will perform meta-analyses to pool results of RCTs. The effect measures will be weighted or standardized mean difference with 95% confidence intervals (CIs) for the continuous outcomes and risk ratio with 95% CIs for the dichotomous outcomes. Subgroup analyses and meta-regression with predefined effect modifiers will be performed to explore the sources of heterogeneity. Where appropriate, we will assess the possibility of reporting bias based on funnel plots and quantitative detection. We will appraise the quality of evidence using the Grading of Recommendations Assessment, Development, and Evaluation system for each outcome.

**Results:**

This study will provide accurate results and balanced inferences on the efficacy and safety of acupuncture and related techniques on T2DM.

**Conclusion:**

This well-designed systematic review will establish high-quality evidence of the efficacy and safety of acupuncture and related techniques for T2DM to facilitate the clinical practice and guideline development.

**PROSPERO registration number:**

CRD42018115639.

## 1 Introduction

### 1.1 Description of the condition

Type 2 diabetes mellitus (T2DM), a metabolic disease manifested as a persistently high level of blood glucose, is caused by the progressive increase in insulin resistance and reduction in insulin secretion.^[1]^ The International Diabetes Federation claimed that approximately 8.8% (415 million) adults suffered T2DM in 2015, and the proportion is predicted to rise to 10.4% (642 million) by 2040.^[2]^ The vascular toxicity of long-term exposure to uncontrolled hyperglycemia increases the risk of a variety of diabetic complications.^[3]^ Cardiovascular diseases are the primary macrovascular complications of T2DM and the leading cause of mortality. A registry study found that the risk of hospitalization owing to heart failure increased by 33% in patients with T2DM.^[4]^ Diabetic patients are also prone to develop microvascular complications, of whom approximately 35% will finally develop diabetic nephropathy,^[5]^ 12.3% diabetic retinopathy,^[6]^ and 29.2% diabetic peripheral neuropathy.^[7]^ Besides, the annual health expenditure by diabetic patients of different sexes and ages ranged from 612 to 1099 billion, which accounted for 11% to 20% of the total health expenditure.^[8,9]^

### 1.2 Description of the intervention

Acupuncture, a popular complementary therapy, was 1st recorded in a traditional Chinese medicine (TCM) classic, The Yellow Emperor's Classic of Internal Medicine, 3000 years ago.^[10]^ Based on TCM theory, acupuncture can be used to treat illnesses by stimulating specific acupoints.^[11]^ Several studies have shown that acupuncture has effects on metabolic and endocrine diseases, such as polycystic ovarian syndrome, thyroid dysfunction, obesity, and T2DM with good safety.^[12,13]^ Currently, available acupuncture and related techniques for these diseases include electroacupuncture (EA), acupressure, moxibustion, warm acupuncture, and transcutaneous acupoint electrical stimulation, and these techniques are commonly applied on the body and auricular acupoints.^[14,15]^

### 1.3 How the intervention might work

Many animal experiments have proved the hypoglycemic effect of acupuncture. A rat study revealed that acupuncture stimulations at Zhongwan (RN12) acupoint increased insulin secretion mediated by activation of opioid receptors.^[16]^ Acupuncture also significantly reduced the insulin resistance in another rat study,^[17]^ which may be associated with the inhibition of free fatty acid production through stimulating cholinergic nerves and activating nitric oxide synthase.^[18]^ A human study also showed that acupuncture improved insulin sensitivity in patients with T2DM, as a result of the reduction in endogenous insulin concentration and the preservation of pancreatic β-cell function.^[19]^ However, the hypoglycemic effects of acupuncture were not observed in rats with type 1 diabetes mellitus.^[16,20]^

### 1.4 Why it is important to perform this review

In the past decade, studies have shown that glycemic control in patients with T2DM has been unsatisfactory.^[21,22]^ Drug dependence and resistance, caused by long-term use and lack of new or alternative treatments, are considered to be the main reasons for this clinical challenge.^[23]^ Equally worrying is the adverse effects of hypoglycemic drugs. For example, alpha-glucosidase inhibitors have a high incidence of gastrointestinal side effects (e.g., abdominal distension and diarrhea) and thiazolidinediones may cause weight gain, limb fractures and even heart failure.^[24,25]^ Acupuncture has potential benefits in efficacy and safety and is thus expected to be a promising complementary treatment for T2DM.^[26]^ Previously, many randomized controlled trials (RCTs) investigated the effects of acupuncture on T2DM, but the results were inconsistent. Some RCTs found that fasting blood glucose (FBG) and hemoglobin A1c (HbA1c) decreased significantly more after acupuncture compared to sham acupuncture,^[27,28]^ but others had negative results.^[29,30]^ Furthermore, multiple observational studies have yielded safety concerns about acupuncture in patients with T2DM. The adverse events included needling pain, hematoma, bleeding, infection, and more severe, necrotizing fasciitis.^[31,32]^ Therefore, it is necessary to perform a systematic review to establish persuasive evidence of efficacy and safety of acupuncture on T2DM.

### 1.5 Objective

The aim of this systematic review is to determine the efficacy and safety of acupuncture and related techniques in patients with T2DM.

## 2 Methods

### 2.1 Study registration

This protocol has been registered in PROSPERO (no: CRD42018115639). The report was guided by the items of Preferred Reporting Items for Systematic Reviews and Meta-Analysis - Protocol (PRISMA-P).^[33]^

### 2.2 Criteria for including studies in this review

#### 2.2.1 Types of studies

We will only include RCTs, the golden standard in therapeutic assessment. The authors should clearly claim that they performed random grouping. Both parallel and crossover trials are eligible, but only the data of the 1st phase will be used in the crossover trials.

#### 2.2.2 Types of participants

Adult patients diagnosed with T2DM according to the American Diabetes Association standards^[1]^ (FBG ≥ 126.1 mg/dL, HbA1c ≥ 6.5%, or random blood glucose ≥ 200.0 mg/dL) are eligible. Trials that recruited patients with type 1 diabetes mellitus, gestational diabetes mellitus, and secondary diabetes mellitus will be excluded. Trials that include patients complicated severe diseases (e.g., heart failure and stroke) are also ineligible.

#### 2.2.3 Types of interventions

##### 2.2.3.1 Experimental interventions

Acupuncture and related techniques on the body and auricular acupoints, including manual acupuncture, EA, acupressure, moxibustion, warm acupuncture, and transcutaneous acupoint electrical stimulation, are eligible. Highly heterogeneous interventions on acupoints or meridians will be excluded, such as laser acupuncture, acupoint catgut embedding, cupping, and Chinese Tuina. The eligible co-interventions include antidiabetic drugs, lifestyle interventions (exercise, diets), whereas Chinese herbs are not allowed.

##### 2.2.3.2 Comparator interventions

The eligible comparators include placebo (i.e., sham acupuncture), antidiabetic drugs, lifestyle interventions, and the combination. Ineligible comparisons include acupuncture vs Chinese herbs, acupuncture vs alternative acupuncture, and acupuncture + nonacupuncture treatment vs alternative nonacupuncture treatment.

#### 2.2.4 Types of outcome measures

##### 2.2.4.1 Primary outcomes

FBG (mg/dL) and HbA1c (%).

##### 2.2.4.2 Secondary outcomes

1. 2-Hour blood glucose (2hBG).
2. Fasting insulin (FINS).
3. Homeostatic model assessment for insulin resistance (HOMA-IR), which is positively correlated with FINS (μIU/mL) and glucose (mg/dL).^[34]^
4. Incidence of new-onset diabetic macrovascular and microvascular complications.
5. Incidence of acupuncture-related adverse effects.

### 2.3 Search methods for identification of studies

#### 2.3.1 Data sources

We will perform literature searches in PubMed, Embase, Cochrane library, and the international registry of RCTs “ClinicalTrials.gov” as of December 3, 2018. We will also trace the reference lists of the published reviews to obtain further eligible trials. An updated search will be performed at the beginning of the data analysis.

#### 2.3.2 Search strategy

As is shown in Table 1, the search fields will include text words and medical subject headings (MeSH), and the keywords will include “diabetes∗,” “T2DM,” “acupuncture,” “acupressure,” “auriculotherapy,” and “moxibustion,” etc. We will not limit the publication language in the search.

**Table 1:**
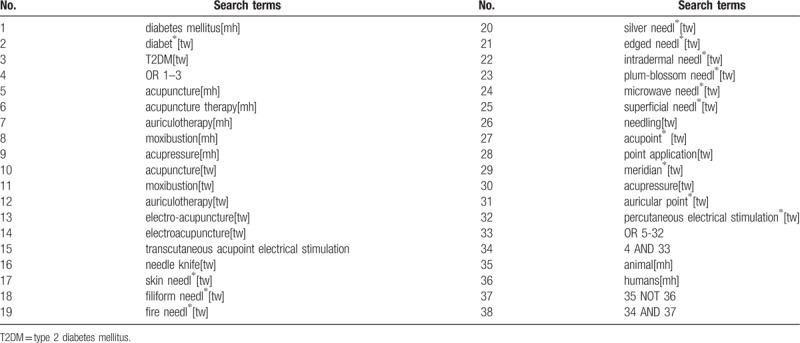
Search strategy utilized for the PubMed database.

### 2.4 Data collection and analysis

#### 2.4.1 Selection of studies

**Figure 1:**
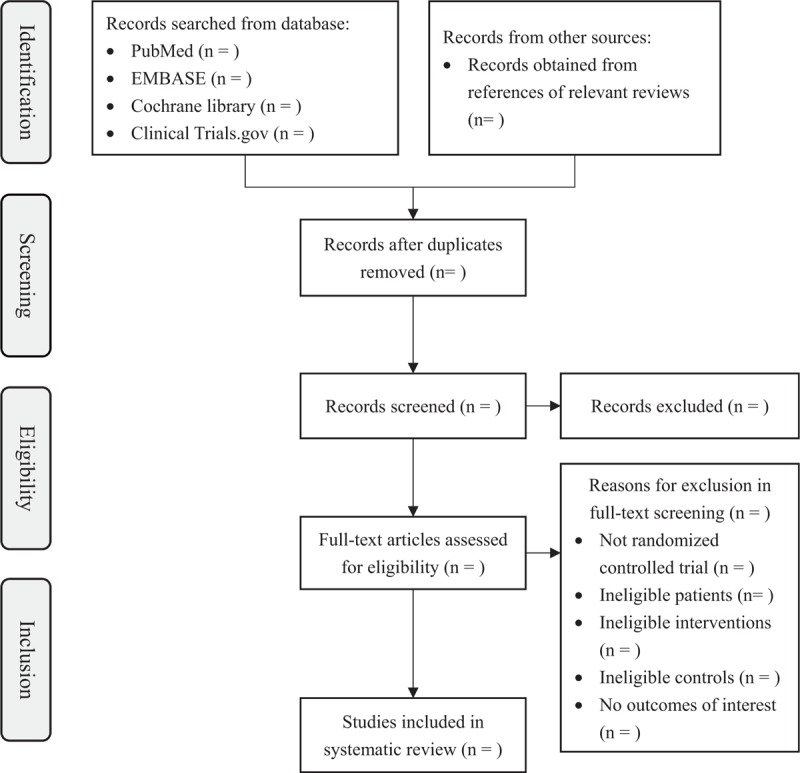
Flow diagram of study selection.

We will import the bibliographies yielded by the search into Endnote X9 (Clarivate Analytics US LLC, Philadelphia) for management and deduplication. Two reviewers, working in pairs, will 1st read the titles and abstracts to exclude the studies with ineligible patients, interventions, controls, or study design. Then, the reviewers will examine the full texts to determine final inclusions according to the full criteria. All the screening processes will be performed independently and in duplicate. The reviewers will resolve any disagreement through discussions or consultations with a senior reviewer. We will give a flowchart to present the detailed screening process compliant with PRISMA-P (Fig. 1).

#### 2.4.2 Data extraction and management

Two reviewers will independently extract the data from the eligible RCTs, and any discrepancy will be settled through discussions or consultations with a senior reviewer. We will extract the following information:

1. Study characteristics: 1st author, publication year, country, language, eligibility criteria, type of acupuncture, details of acupuncture methods (according to the Standards for Reporting Interventions in Controlled Trials of Acupuncture [STRICTA] criteria^[35]^), type of control, number of patients, follow-up time, and dropout rate.
2. Patient characteristics: gender, age, comorbidities, use of antidiabetic drugs, and baseline FBG, HbA1c, and body mass index.
3. Outcome data: mean and standard deviation (SD)/standard error will be extracted for continuous outcomes; the number of events and total population will be extracted for dichotomous outcomes. If a study provides data of multiple follow-up nodes, we will only use the data of the final time point data for data analysis.

#### 2.4.3 Assessment of risk of bias

We will use the Gordon H. Guyatt's revision of Cochrane risk of bias tool for the assessment.^[36,37]^ Seven aspects of risk of bias will be assessed, including random sequence generation, allocation concealment, blinding of patients and caregivers, blinding of outcome assessment, data completeness, selective outcome reporting, and other bias (e.g., extremely imbalanced baselines between the groups). In contrast to the original tool, this revision requires the reviewers to judge “unclear” items as “probably yes” or “probably no” based on the relevant contexts in the article.^[37]^ We will finally classify the overall risk of bias into three levels: low (all items have a low or probably low risk of bias), moderate (at least 1 item probably has a high risk of bias), and high (at least 1 item definitely has a high risk of bias). The assessment will be carried out by 2 reviewers, and any discrepancy will be resolved through consultation with a senior reviewer.

#### 2.4.4 Measures of treatment effect

We will use mean difference (MD) with 95% confidence intervals (CIs) as the effect measure for continuous outcomes. For dichotomous data, we will use risk ratio (RR) with 95% CIs as the effect measure.

#### 2.4.5 Dealing with missing data

For continuous variables, we will estimate the missing mean and SD of change from baseline using the data at baseline and the last follow-up by the Cochrane Collaboration method.^[36]^ Data described as median and range or interquartile range will be converted to mean and SD by the Wan method.^[38]^ We will also try to contact authors to request for the missing data that cannot be imputed or inferred from the article.

#### 2.4.6 Assessment of heterogeneity

We will conduct the Cochran *Q* test and estimate the *I*^2^ statistics to detect the heterogeneity for each outcome.^[36]^ A *P*-value <.1 or *I*^2^ > 50% indicates significant heterogeneity; otherwise, heterogeneity is acceptable.

#### 2.4.7 Assessment of reporting biases

For the outcomes in which at least 10 RCTs are included, we will assess the possibility of reporting bias. The funnel plots will be used to assess asymmetry visually. The Egger test^[39]^ (for continuous data) and the Harbord test^[40]^ (for dichotomous data) will be used for the quantitative assessment. A *P* < .05 indicates a significant publication bias.

#### 2.4.8 Data synthesis

In the meta-analysis of continuous outcomes, we will pool the weighted MD (WMD), or standardized MD (SMD) if the units cannot be unified, by the inverse variance method. An SMD < 0.5 will be generally considered to be a small effect size, 0.5 to 0.8 to be moderate, and >0.8 to be large.^[41]^ In the meta-analysis of dichotomous outcomes, we will pool RR by the Mantel–Haenszel method. When necessary, the logRR/WMD/SMD and standard error will be calculated and pooled by the inverse variance method. Random effects model will always be used to combine the data independent of the magnitude of heterogeneity. We will conduct a narrative summary when the results are inappropriate to be pooled due to the substantial clinical or methodologic heterogeneity. We will use forest plots to present the results of the meta-analyses. The statistical analysis will be performed using RevMan v5.3.5 (The Cochrane Collaboration, The Nordic Cochrane Centre, Copenhagen, Denmark) and Stata v14.0 (StataCorp Lp, TX).

#### 2.4.9 Subgroup analysis and investigation of heterogeneity

If data are available, we will perform subgroup analyses and meta-regression to explore the sources of heterogeneity. Four independent variables are predefined: type of acupuncture (skin-pierced acupuncture vs others); type of control group (antidiabetic drugs vs nondrug controls); risk of bias (high vs moderate vs low); follow-up period (≥8 vs <8 weeks). We will also consider performing post-hoc subgroup analyses and meta-regression for other independent variables if necessary.

#### 2.4.10 Sensitivity analysis

To evaluate the robustness of the pooled results, we will perform sensitive analysis by including only RCTs with a low or moderate risk of bias and using an alternative effect model (random effects model vs fixed-effect model).

#### 2.4.11 Summary of evidence

We will rate the quality of evidence for each outcome using the Grading of Recommendations Assessment, Development, and Evaluation (GRADE) instrument.^[42]^ The quality of evidence will initially be high in all outcomes but will be degraded to be moderate, low, or very low, if there are weaknesses in risk of bias, heterogeneity, indirectness, imprecision, and publication bias. Two reviewers will independently perform this process, and a 3rd reviewer will join if necessary.

### 2.5 Ethics and dissemination

Ethics approval is not required in this study because no individual data from participants will be involved. We aim to publish this systematic review on a peer-reviewed journal.

## 3 Discussion

A few systematic reviews discussed the topic of acupuncture for T2DM previously.^[43–45]^ However, the targets of these reviews are substantially different from ours, one of which only included herbal acupuncture,^[43]^ one only included manual acupuncture,^[44]^ and the other one only focused on diabetic gastroparesis.^[45]^ Except for the 1st one, all reviews did not assess the outcomes related to the hypoglycemic effects (e.g., FBG, HbA1c, and FINS). These reviews also have substantial methodologic limitations, such as lack of detailed descriptions on the acupuncture methods according to STRICTA criteria,^[43–45]^ high heterogeneity without explanation,^[43–45]^ and no assessment of the quality of evidence.^[43,44]^

In contrast to the published systematical reviews, our review will include all acupuncture and related techniques and focus on outcomes reflecting the hypoglycemic effects and the safety. There will be several methodologic advantages in our study. First, we will judge “probably yes” and “probably no” for the items with “unclear” risk of bias to achieve a more transparent assessment. Second, we will test 4 predefined hypotheses in the subgroup analysis and meta-regression based on the consideration of clinical and biologic mechanisms, which will enhance the credibility of the results.^[46]^ Third, we will use the GRADE instrument to critically assess the quality of evidence of each outcome to make objective conclusions.

A potential limitation in our review is that we will include RCTs with various characteristics (e.g., different types of acupuncture and control and inconsistent length of follow-up), which may inevitably lead to heterogeneity. Although we plan to perform relevant subgroup analysis and meta-regression, the source of heterogeneity may be hard to be fully elucidated due to insufficient data. Besides, we intend to only search 4 central English databases, which means that the RCTs only recorded in the Chinese databases will be omitted and seems to cause selection bias. Nevertheless, some methodologists have deemed those Chinese RCTs extremely unreliable, as most of them only describe “random grouping” without further information about randomization and lack of trial registration and monitoring.^[47,48]^ Therefore, the exclusion would be helpful to reduce the overall risk of bias rather than leading to disadvantages. We believe that this well-designed systematic review will establish high-quality evidence to facilitate clinical practice and guideline development of acupuncture for T2DM.

## Author contributions

**Conceptualization:** Xu Zhou

**Funding acquisition:** Xu Zhou

**Investigation:** Weifeng Zhu, Dongmei Yan, Heyun Nie, Xiaofan Chen

**Methodology:** Meilu Liu, Jianrong Chen

**Writing – original draft:** Meilu Liu, Jianrong Chen.

**Writing – review & editing:** Qing Ren, Xu Zhou.

Xu Zhou orcid: 0000-0002-9541-4796.
